# When Choice Makes Sense: Menthol Influence on Mating, Oviposition and Fecundity in *Drosophila melanogaster*

**DOI:** 10.3389/fnint.2016.00005

**Published:** 2016-02-22

**Authors:** Dehbia Abed-Vieillard, Jérôme Cortot

**Affiliations:** ^1^Centre National de la Recherche Scientifique, UMR6265 Centre des Sciences du Goût et de l’AlimentationDijon, France; ^2^Institut National de la Recherche Agronomique, UMR1324 Centre des Sciences du Goût et de l’AlimentationDijon, France; ^3^UMR Centre des Sciences du Goût et de l’Alimentation, Université de BourgogneDijon, France

**Keywords:** oviposition preference, courtship, mating, fecundity, *Drosophila melanogaster*, menthol, choice

## Abstract

The environment to which insects have been exposed as larvae and adults can affect subsequent behaviors, such as mating, oviposition, food preference or fitness. Experience can change female preference for oviposition, particularly in phytophagous insects. In *Drosophila melanogaster*, females avoid laying eggs on menthol rich-food when given the choice. Exposure to menthol during larval development reduces this aversion. However, this observation was not reproduced in the following generation. Recently, we have shown that oviposition-site preference (OSP) differs between wild-type *D. melanogaster* lines freely or forcibly exposed to menthol. After 12 generations, menthol “forced” lines still exhibit a persistent aversion to menthol whereas ‘free-choice’ lines show a decreased aversion for menthol rich-food. Here, we compare courtship behavior, mating and female fecundity in “forced” and “free-choice” lines, raised either on menthol rich-food (Menthol-lines) or on menthol-free food (Plain-lines). “Forced” males did not discriminate between decapitated virgin females of the two lines. They courted and mated with intact females of both “forced” lines in a comparable rate. However “forced” M-line males did mate significantly more rapidly with “forced” M-line females. In the “free-choice” procedure, P-line males show a similar pattern as “forced” males for discrimination ability and courtship. M-line males courted significantly more M-line females. Both ‘free-choice’ lines males mated significantly more with females of their own line. Female fecundity was assessed during 10 days in ‘free-choice’ lines. Menthol-line females laid more eggs during the first 4 days than female Plain-lines and parental control-line. The total number of eggs laid during the first 10 days of female adult life is comparable in M-line and parental control line. However, Menthol-line females laid eggs earlier than both parental control and Plain-lines. Our findings show that in *D. melanogaster*, as for OSP, mating and fecundity are more rapidly influenced when flies have a choice between alternative resources compared to flies permanently exposed to menthol.

## Introduction

Choosing a suitable oviposition site is a key decision for egg-laying animals such as insects. On this decision depend the survival and even the fitness of the future generation. This is particularly important for animals that remain in the vicinity of or/and feed on the oviposition sites. Numerous factors can affect female decision: food quality and abundance, density of eggs, eggs load, age (Minkenberg et al., 1992 and reference therein), presence of predators, surrounding vegetation, chemical cues of conspecifics (Bentley and Day, 1989; Mokany and Shine, 2003), social learning (Sarin and Dukas, 2009), or prior experience with plants or plant stimuli (Papaj and Prokopy, 1989). Flies are able to oviposit on a wide range of fruits (Atkinson and Shorrocks, 1977) and host selection can be a genetically complex phenomenon (Jaenike, 1986). In *Drosophila melanogaster*, female oviposition preference can be influenced by social composition, exposure to temperature, surface texture or color of the substrate (Volpe et al., 1967; Fogleman, 1979; Takamura and Fuyama, 1980; Battesti et al., 2015). Similarly, prior exposure to particular food or compounds can also influence female choice (Jaenike, 1983; Abed-Vieillard et al., 2014; Flaven-Pouchon et al., 2014). Exposure to a particular odor can enhance the preference or reduce the aversion to other odors, referred by Jaenike (1983) as cross-induction (Barron and Corbet, 2000). Selection studies on behavioral traits such as oviposition or mating had been conducted. Fogleman (1979) failed to establish strains for low and high temperature preference for oviposition after eight generations. Artificial selection on grape or quince for oviposition site preference did not induce a change in preference in *D. melanogaster* (Soto et al., 2014). Flies forcibly raised on menthol-rich food for more than 30 generations did not change their aversion to this compound. However, aversion to lay eggs on menthol-rich food can significantly decrease after only 12 generations in free-choice line flies (Abed-Vieillard et al., 2014). These results show that the nature of the compound is an important factor for selection (Jaenike, 1983).

Speciation in phytophagous insects and particularly in the *Drosophila* group has been intensively studied (Coyne and Orr, 2004; Matsubayashi et al., 2010). Adaptation to a new environment or colonization of novel host plants may lead to sexual isolation (Etges, 1990; Feder et al., 1994). Ethological isolation (sexual or behavioral) is one of the reproductive isolation mechanisms in animals, which can be defined as the deviation from random mating in mated individuals, “individuals will avoid mating with those of another strain, race or species” (Gilbert and Starmer, 1985). Besides the genetic background, external factors such as temperature, diet, density or previous experiences, can potentially act on reproductive isolation (Spieth and Ringo, 1983; Sharon et al., 2010; Najarro et al., 2015).

Pre-exposure to peppermint oil or to menthol (its main component) can reduce aversion in adult flies (Thorpe, 1939; Barron and Corbet, 1999a,b, 2000). We recently compared the oviposition site preference of wild-type *D. melanogaste*r lines freely or forcibly exposed to menthol-rich food. After 12 generations, oviposition site preference differs between the two lines (Abed-Vieillard et al., 2014). The aim of this study was to investigate whether menthol can influence mating and fecundity in *D. melanogaster* by comparing the “free-choice” lines and the “forced” lines. Our results suggest that when females can freely choose between two egg-laying sites, their choice can influence subsequent behaviors in the offspring.

## Materials and Methods

### Flies

Wild-type Dijon2000 strain (Di2) *D. melanogaster* Meigen established in 2000 were used. Flies were raised on a yeast– cornmeal–agar medium and kept at 25 ± 0.5°C with 65 ± 0.5% humidity in a 12-h light: 12-h dark cycle.

### Menthol and Food Preparation

Plain food (P-Food) and Menthol enriched food (M-Food) diets for adult *D. melanogaster* were prepared as described in Abed-Vieillard et al. (2014). A 250 mg/ml solution was prepared by dissolving menthol (Pure racemic menthol, M0321, TCI, Japan) in 90% (v/v) ethanol and kept at 4°C until used. Menthol solution was added to fresh lab food = menthol-food or (M-food). Three concentrations of menthol-food (0.01, 0.1, and 0.5%) were used. A similar volume of ethanol (90% v/v) was added to the control diet menthol-free food (Plain-food: P-food).

### Exposure Procedures

Even if food enriched with 0.1% of menthol elicited an aversion, females still laid eggs on it allowing the establishment of menthol lines. “Choice” and “Forced” lines were established as described in Abed-Vieillard et al. (2014). In summary, the “forced” procedure consisted to raise individuals during their complete development, generation after generation, either on M-food (0.1% menthol; Forced M-line) or P-Food (without menthol; Forced P-Line). The “choice” procedure consisted in keeping the progeny left during a dual choice test (P-food versus M-Food) either on M- or on P-food, generation after generation on that food. Parental “control line,” strains unexposed to menthol, were kept in similar laboratory conditions.

### Behavioral Tests

All flies were isolated 0–4 h after emergence under CO_2_ anesthesia. For courtship tests, male flies held individually and females, held in groups of five, were kept in fresh glass control food vials for 5 days before testing. For oviposition tests, males or females were held in groups of 25 in similar laboratory conditions. All tests were performed in a room at 25°C ± 0.5% with 65 ± 0.5% humidity.

### Oviposition Preference and Survival

We performed 4-choice tests using 4 egg-laying sites filled respectively with 0, 0.01, 0.1, and 0.5% menthol food. Twenty-five virgin females and 25 males (parental control-line individuals, 4–5-day-old, previously CO_2_-anesthetized) were introduced in the test device (described by Abed-Vieillard et al., 2014) during 12 h at 25°C. The number of eggs laid on each site was counted. In order to assess the survival rate, we allowed larvae to fully develop at 25°C. Adults were counted for each site. Sites with less than 10 eggs were discarded as for 0.5%, menthol concentration where females rarely oviposited. Twenty-three replicates of four-choice oviposition preference tests were performed.

### Courtship Assays

Two kinds of experiments were performed (i) with decapitated females to measure male ability to discriminate between females of P and M-lines and (ii) with intact females to measure mating success.

#### Male Discrimination

Tests were performed using the same protocol as described by Houot et al. (2010). A watch glass was used as courtship observation chamber (1.6 cm × 1.6 cm × 1.6 cm). Tester males, whose sexual response to target flies was measured, were individually aspirated (without anesthesia) under the observation chamber and let 5 min to habituate. Two female target flies (one female for each line) were introduced and the 10 min observation period started. To distinguish females, one millimeter at the end of a wing of one of the females was cut. To characterize male discrimination, we measured the proportion of time spent by tester males in actively courting (wing vibration, licking, and attempting copulation; total = courtship index, CI) each target female. For each male, we obtained two values corresponding to the CI directed to each target female (CIP: CI for female of the P-Line and CIM: CI for female of the M-line). Tests were carried out under a dim red light (25W with a Kodak Safe- Light Filter n°1) to remove all visual stimuli. Target females were decapitated to remove most acoustic and behavioral signals.

#### Courtship and Mating

For the mating experiment, each tester male was paired with a virgin female, for at most 1 h. We measured the CI during the first 10 min, the latency to copulate (time in min from the introduction of the female into the chamber until copulation), the duration of copulation (time in min from the copulation onset until disengagement), and the frequency of copulating pairs for each cross combination.

Tests were performed for the forced-lines and the choice-lines. Flies were paired as follows: Male M-Line × Female M-line; Male M-Line × Female P-line; Male P-line × Female P-Line and Male P-Line × Female M-Line.

### Female Fecundity

Abed-Vieillard et al. (2014) noticed a significant increase of the number of eggs laid by females in the choice-procedure compared to parental control-lines. In this study, female oviposition preference was checked once from day 1 to day 5. They showed that Choice P-line and M-line females laid 2–4 times more eggs than the Control-lines. In order to assess if this increase reflects an increased fecundity of the choice-lines females, 20 12 h-old virgin flies (10 males and 10 females) were introduced in a oviposition test chamber containing two egg-laying sites, one filled by P-Food and one by M-Food. The parental control line was tested in a similar way. Females were allowed to lay eggs for 24 h. Then the two sites were removed and replaced by new fresh ones. The tests lasted 10 days. Eggs on each site were counted immediately or frozen until counting. At least 10 replicates were made for each line.

### Statistics

All data were transferred to Prism 5.0d (GraphPad) for statistical analysis except for the ANCOVA [XLSTAT 2012 software (Addinsoft, XLSTAT 2012, Data analysis and statistics with Microsoft Excel, Paris, France)]. Pairs of data were analyzed using the Mann–Whitney test (courtship assays) or Wilcoxon signed ranked test (male discrimination). Groups of more than 2 data sets were compared by Kruskal–Wallis (KW) test followed by a Dunn’s multiple comparison test. We used an ANCOVA to compare the fecundity of the tree-line females (referred as cumulated number of eggs per day). The number of eggs were considered as dependent variables, whereas ≪line type≫ (control, P-, M-choice lines) was considered as a qualitative independent variable and ≪day≫ as an independent quantitative variable. When the ANCOVA result was significant, we used the Tukey test to compare food type effect.

## Results

### Oviposition Site Preference

**FIGURE 1:**
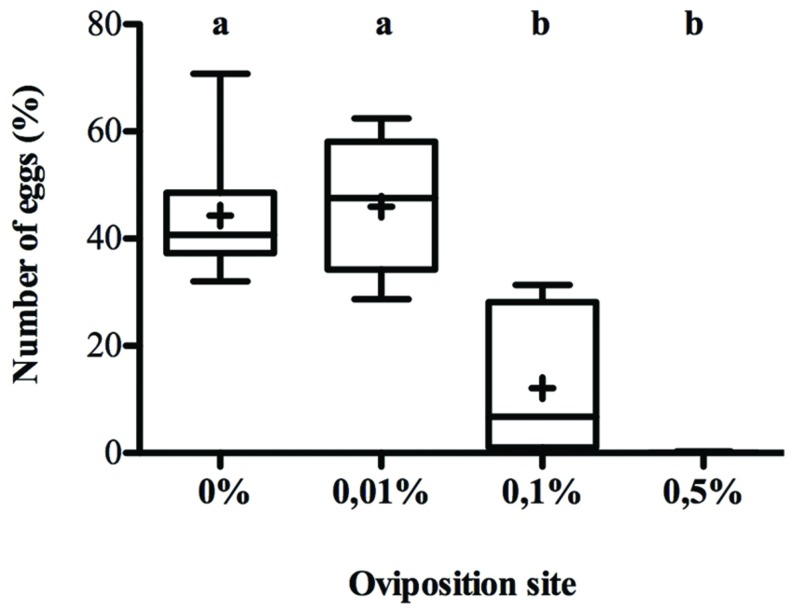
***Drosophila melanogaster* oviposition preference on menthol-enriched food.** Females were given a choice to lay eggs on four sites containing food enriched with 0, 0.01, 0.1, or 0.5% menthol during 12 h. Eggs were then counted for each site and the percentage of eggs in each site is given in the *y*-axis. Data are represented both with their mean (noted by +) and by box-and-whisker plots (the bars represent the first and third quartiles (Q1 and Q3) with the horizontal band inside the bar indicating the median value; the whiskers indicate the minimum and maximum of all of the data. Different letters indicate a significant difference (Kruskal–Wallis, *K*_4__df_ = 54.76, *p* < 0.00001) (*N* = 23).

Female oviposition preference response to menthol-rich media were inversely correlated to menthol concentration (*K*_3__df_ = 54.76, *p* < 0.0001) (**Figure 1**). *D. melanogaster* females laid eggs in a similar way in menthol-free and 0.01% menthol enriched food (55% over 46% respectively). When menthol concentration increased, females displayed a strong aversion and only about 10% of the eggs were laid in the 0.1% menthol site whereas females rarely laid eggs on 0.5% menthol.

### Male Discrimination and Courtship Behavior Against Decapitated Females

**FIGURE 2:**
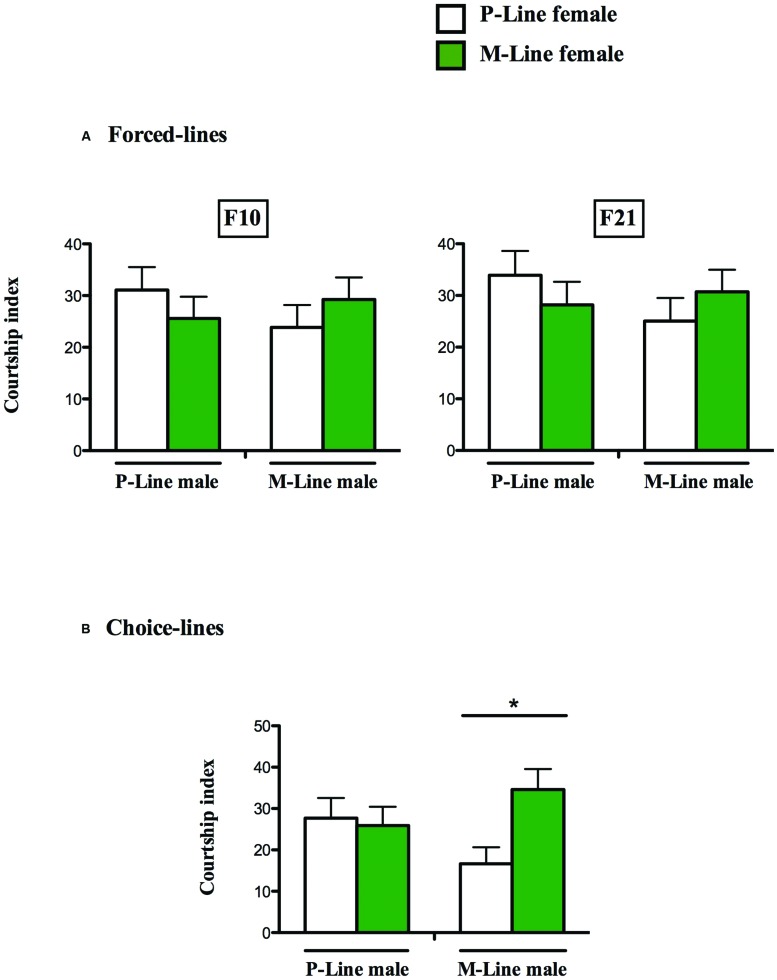
**Male discrimination in two differently exposed lines.** Mean (±SEM) male courtship index to P-line (open bars) and M-line (green bars) target females. A pair of decapitated female flies (a P-line and a M-line female) was simultaneously presented to a single tester male, under red light, during a 5-min period. For each exposed line, Forced-line **(A)** and Choice-line **(B)**, the male ability to discriminate and court the two target females is shown above each pair of bars (^∗^*p* < 0.05). **(A)** *N* = 32 **(B)** *N* = 56–65.

In the 10th generation forced-line, when given a choice between P- and M-line virgin decapitated females, P- and M-line males courted the females in a similar way (CIP = 31.07 ± 4.45 and CIM = 25.58 ± 4.19 for P-Male; CIP = 23.85 ± 4.34 and CIM = 29.25 ± 4.26 for M-Male) (Wilcoxon test, *U* = 162, *p* = 0.500, *n* = 55 and *U* = -170, *p* = 0.428, *n* = 52 respectively). The same results were obtained with individuals of the 21st generation (CIP = 33.92 ± 4.68 and CIM = 28.20 ± 4.45 for P-Male; CIP = 25.04 ± 4.48 and CIM = 30.71 ± 4.28 for M-Male) (Wilcoxon test, *U* = 144, *p* = 0.502, *n* = 52 and *U* = -167, *p* = 0.423, *n* = 51 respectively for P-males and M-males (**Figure 2A**).

In the 10th generation Choice-lines, P-line males courted in a similar manner P and M-line females (CIP = 27.69 ± 4.89 and CIM = 23.91 ± 4.51) (Wilcoxon test, *U* = 22, *p* = 0.844, *n* = 32). Unlike P-males, M-Line males spent significantly more time courting M-line females than P-line females (CIP = 16.85 ± 4.00 and CIM = 34.59 ± 4.96) (Wilcoxon test, *U* = -236, *p* = 0.028, *n* = 32) (**Figure 2B**).

### Courtship and Mating

#### In Forced-Lines

**FIGURE 3:**
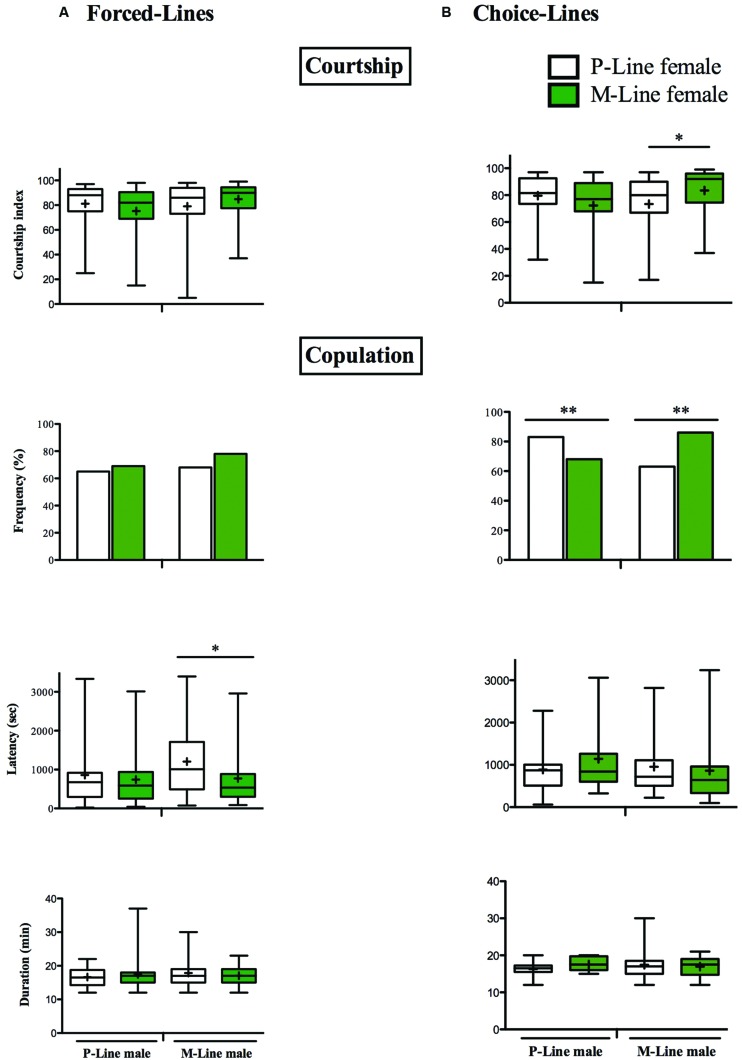
**Courtship behavior and mating in two differently exposed lines.** Individual 5 day-old males were paired with an intact female of the P-line or the M-line. The box-and-whisker plots show the male courtship index, the latency and the duration of mating. The bar graphs show the frequency of mating. The male courtship index (CI) to the female is measured for 10 min. The frequency of mating, the copulation latency (copulation onset, in sec) and the copulation duration (in min) were measured for 60 min. P-line females: open bars and open boxes, M-line females: green bars and green boxes. **(A)** Forced-line, **(B)** Choice-line (^∗^*p* < 0.05; ^∗∗^*p* < 0.01). **(A)** *N* = 12–17 **(B)** *N* = 45–49. For more information, refer to **Figure 1**.

Males of both P- and M-lines courted in the same way P- and M-females (Mann–Whitney test, *p* = 0.218 and *p* = 0.175 respectively for P-male and M-male). Among the different parameters measured, we only found a significant difference in the latency of mating, M-males mating faster with M-females than with P-females (respectively 771.1 s ± 124.5 and 1207 s ± 155.5; Mann–Whitney test, *p* = 0.031). Duration of mating and mating success were the same for all the inter-line combinations (**Figure 3A**).

#### In Choice-Lines

P-males displayed a similar intensity of courtship in front of P and M-females (CIP = 81.16 ± 2.68 and CIM = 78.18 ± 5.19; Mann–Whitney test, *p* = 0.210). Latency and duration of mating were similar in both inter-line combinations (Mann–Whitney test: *p* = 0.766 and *p* = 0.314 respectively for mating latency and mating duration) (**Figure 3B**). However, males of the P-line mated significantly more with P-females than with the M-females (two-tailed Fisher exact test, *p* = 0.020). M-males courted significantly more M-females than P-females (respectively CIM = 83.48 ± 3.92 and CIP = 73.42 ± 5.19; Mann–Whitney test, *p* = 0.046). Whilst mating latency and duration were the same in front of P and M-females (Mann–Whitney test: *p* = 0.378 and *p* = 0.808 respectively), M-males mated significantly more with the latter (two-tailed Fisher exact test, *p* = 0.0003).

### Influence of Menthol on Female Fecundity

**FIGURE 4:**
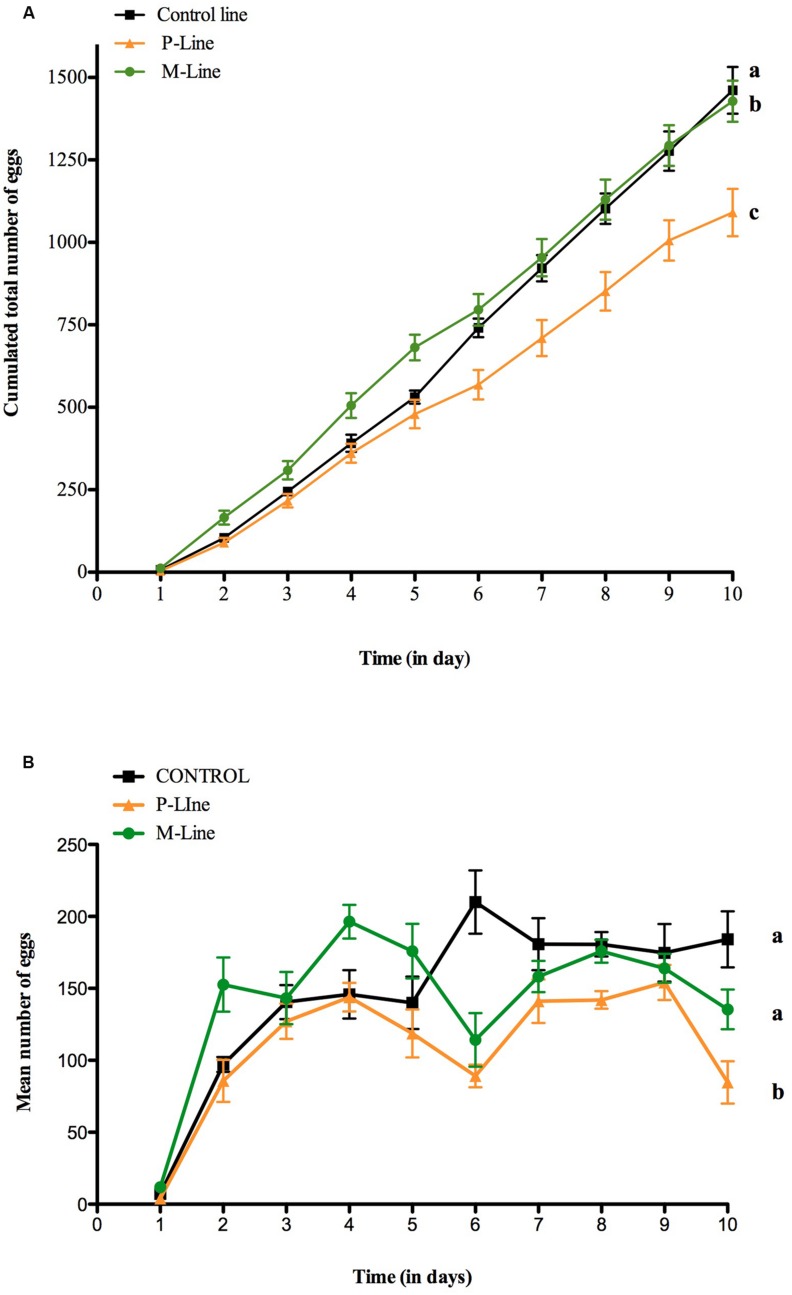
**Fecundity of *D. melanogaster* females during the first 10 days after emergence in an oviposition two-choice test.** Control line (C-line) and the two choice-lines (P- and M-line) were tested. Mean (±SEM) of eggs laid by females of the three lines on two oviposition sites during 10 days after emergence. **(A)** Cumulated number of eggs and **(B)** Number of eggs laid daily. Twenty flies (10 females and 10 males) were placed in an arena containing two oviposition sites, one filled with plain-food and one filled with 0.1% menthol enriched food. Females could lay eggs for a 24 h period then the two sites were replaced by new ones. The total number of eggs laid on the two sites was counted daily. *N* = 8 for each line. Different letters indicate statistical difference [ANCOVA: *F*_3,236_ = 916.05, *p* < 0.0001 for **(A)** and, *F*_3,236_ = 28.902, *p* < 0.0001 for **(B)**].

Females raised on menthol began to lay eggs earlier than those raised on Control and P-food (ANCOVA, *F*_3,236_ = 916.05, *p* < 0.0001) (**Figure 4A**). They laid an increasing number of eggs during the first days after emergence reaching a maximum at day 4 (mean = 196.4 eggs ± 11.69), followed by a decrease in day 5 and 6 before increasing again during the last 3 days (**Figure 4B**). P-line females showed a similar pattern but laid significantly less eggs than M-line females (ANCOVA, *F*_3,236_ = 28.902, *p* < 0.0001). Control-line females reached the maximum of eggs laid 2 days later than both choice-lines. After 7 days, the total number of eggs was similar between Control-line and M-line females (**Figure 4A**). The total number of eggs laid by females after 10 days of experiment was similar for the Control- and the M-Line but was significantly different for the P-Line. Whilst total fecundity was not affected, females of choice-line and particularly M-line females laid eggs precociously compared to control-line females.

### Influence of Menthol on Survival Rate in Choice-Lines

**FIGURE 5:**
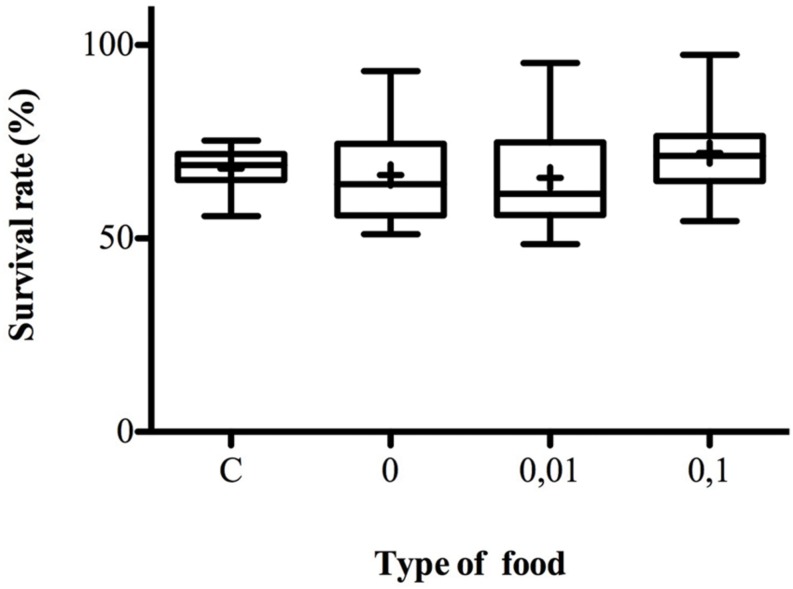
**Survival rate on control and menthol-enriched food.** The box-and-whisker plots show the survival rates on different types of food, C, control food, 0%, no menthol plain-Food, 0.01, 0.01% menthol-enriched food, 0.1, 0.1% menthol-enriched food. Females were given the choice to lay eggs on four different oviposition sites with increased menthol concentration ranging from 0 to 0.5%. For each site larvae were raised at 25°C until complete development. For Control-line survival rate is measured in a multiple choice assay with four sites filled with control food and flies from parental lines never exposed to menthol. Adults were counted at emergence and survival rate was measured. *N* = 10–16. For more information, refer to **Figure 1**.

Larvae fully developed into adulthood in a similar way in control media and media supplemented with 0.01 or 0.1% of menthol (*K*_3__df_ = 2.772, *p* = 0.4282) (**Figure 5**). Menthol at the tested concentrations did not delay nor compromise *D. melanogaster* development.

## Discussion

### Menthol, Courtship and Behavioral Reproductive Isolation

Courtship behavior in *D. melanogaster* involves multimodal sensory signals (visual, olfactory, acoustic, and tactile) (Spieth and Ringo, 1983; Yamamoto et al., 1997; O’Dell, 2003). In our study, differences were observed between the forced and the free-choice lines after ten generations. While ‘Forced-line’ males of both lines courted and mated similarly with P and M-line females even after 21 generations, showing no ethological reproductive isolation, ‘Free-choice line’ males mated significantly more with females of their own line. But only choice M-line males were able to discriminate and court more M-females. Like for oviposition, only the choice procedure induced an ethological isolation.

Influence of larval diet on mating preference has been reported in *Drosophila* species (Dodd, 1989; Etges, 1992; Brazner and Etges, 1993; Pavković-Lucić, 2009; Sharon et al., 2010) with different conclusions. *D. pseudoobscura* flies showed a reproductive isolation between starch-reared and maltose-reared populations (Dodd, 1989). Similarly, an inbred strain of *D. melanogaster* mated assortatively based on the diet of previous generations (“molasses medium” versus “starch medium”) (Sharon et al., 2010; Najarro et al., 2015). Interestingly, mating preference appeared after only one generation of growth in these different media and lasted for more than 37 generations (Sharon et al., 2010). However Najarro et al. (2015) were unable to generate assortative mating with an outbred strain. When flies were reared or experienced the rearing media in a comparable way as our “forced” lines, no ethological isolation was found (Pavković-Lucić, 2009). The discrepancy between these results is most probably due to the type of medium used. When assortative mating occurred, the strains were raised on different types of food. The microbiome of the two diets were different. No ethological isolation was found when fruit extracts or chemical components (as menthol or ethanol) were added to control food, without changing the type of food. The microbiome was probably not or only slightly affected. When assortative matings occurred, the studies clearly showed the implication of extracellular microbes in this reproductive isolation (Sharon et al., 2010). Cuticular hydrocarbon composition has been shown to change according to diet (Etges et al., 2006; Fedina et al., 2012). The modification on the microbiota led to a modification of the epicuticular hydrocarbon profile of the flies (Sharon et al., 2010).

In our experiment only “Free-choice” M-line males were able to discriminate decapitated or intact females of their own line. Males relied most certainly on chemical cues as our experiments were performed under red light with decapitated females. A GC analysis of female hydrocarbons of both P- and M-choice lines revealed that M-line females produced significantly more 7,11-heptacosadiene, a female specific hydrocarbon, than P-lines females (Supplementary Figure S1). 7,11-heptacosadiene has been reported as the most potent aphrodisiac for males (Antony et al., 1985; Wicker-Thomas, 2007 and reference therein). Although other compounds such as 7,11-nonacosadiene and 7-pentacosene play a role in sex recognition, we did not find any difference between the choice-lines. Differences in the amount of 7,11-heptacosadiene in M-line females might explain why M-males courted more M-females when given the choice between P and M-females. We cannot exclude that some ‘menthol odor’ was still present on the flies raised on menthol and that males used this cue for discrimination. However, flies (males and females) were kept in standard medium (without menthol) during the first days of their adult life. Thus, when tested, the amount of menthol adsorbed in the cuticle of flies should be very low. Our GC-analysis setting for hydrocarbons did not allow us to detect or quantify menthol. Several authors reported an extinction of the response to menthol a few hours after having been exposed (Thorpe, 1939; Barron and Corbet, 2000). If so, difference in sensitivity (threshold) may explain the ability of free-choice male to recognize females of their own line, as they were in contact with menthol during their development.

### Menthol and Oviposition

For generalist insect species, such as *D. melanogaster*, selecting an appropriate oviposition site is essential for progeny survival and fitness. Visual, olfactory and gustatory cues are involved (Renwick and Chew, 1994). In *Drosophila*, Jaenike (1982) proposed two phases: settling and oviposition. To settle, females use visual and olfactory cues. Before laying eggs, female flies evaluate the composition of the medium through olfactory receptors in the antenna (Dweck et al., 2013) and gustatory receptors present in their proboscis and ovipositor (Yang et al., 2008). Then, concentration of specifics chemicals can induce or prevent oviposition (Depetris-Chauvin et al., 2015). Egg-laying preference is primarily relayed through gustatory neurons (Joseph et al., 2009).

When given the choice to lay eggs on menthol-rich food females *D. melanogaster* showed clear avoidance as concentration increased. This natural repulsive effect has been shown in other *Drosophila* species (Jaenike, 1982) and most generally in arthropods (Kumar et al., 2011 and reference therein). Pre-exposure to peppermint or menthol can reduce significantly the aversion (Barron and Corbet, 1999a) and can explain the shift from aversion to indifference for a 0.1% menthol substrate in the choice M-line females (Abed-Vieillard et al., 2014). Menthol can be perceived through olfactory and gustatory modalities. However, the precise mechanisms for menthol perception in *Drosophila* remain unclear. No olfactory receptor has yet been identified (Turner and Ray, 2009; Silbering et al., 2011; Münch and Galizia, 2015). In mammals, menthol can activate two transient receptor potential channels: the TRP melastatin-eight TRPM8, which serve as a cold sensor and the TRPA1. But there is no evidence of such a function in *Drosophila* TRPM8 (Fowler and Montell, 2013; Saito and Tominaga, 2015, for review) and TRPA1 is insensitive to menthol in non-mammalian species (Xiao et al., 2008). In *D. melanogaster*, thermotaxis response to cool temperatures requires the function of the TRP and TRPL channels (Bellemer, 2015 for review). Besides serving as a cold sensor, TRPL is involved in gustatory responses. It is involved in detection of aversive compounds and is expressed in bitter neurons in the labellum (Zhang et al., 2013). Menthol has been reported to inhibit the TRPL channel (Parnas et al., 2009). As for camphor (Zhang et al., 2013), it is possible that long term feeding on menthol can affect TRPL protein levels, making a menthol rich-medium more acceptable for feeding and oviposition in the M-choice line. A limited number of olfactory pathways seem to be involved in oviposition site selection and female preference can be mediated via only a single olfactory channel in several *Drosophila* species (Dekker et al., 2006; Yang et al., 2008; Herrero, 2012; Dweck et al., 2013; Linz et al., 2013; Keesey et al., 2015).

### Menthol and Fecundity

According to the preference-performance hypothesis, females should choose the site were their offspring fare best. A 0.1% menthol concentration did not impair larval development and adult survival, but a 0.15% concentration reduced fly survival (Barron and Corbet, 1999b). Food supplemented with 0.05% of peppermint is lethal for *D. recens* adults (Jaenike, 1982) and high concentration (more than 0.5%) of peppermint oil is toxic for *D. melanogaster* larva (Thorpe, 1939). Here we showed that a 0.1% menthol concentration did not impair *Drosophila* development and that females of the Choice M-line laid eggs earlier than Control-line females. These results are consistent with a previous study (Abed-Vieillard et al., 2014) where females of the Choice-line were only tested for oviposition from day 1 to day 5 after hatching. Here we show that the increased number of eggs during the first days of adult life was not due to an increase in female fecundity but to precocious egg laying. However, we cannot exclude that this phenomenon may be due to menthol-induced hastened female maturity or oogenesis.

The genetic basis for oviposition site preference and the decision to oviposit has been investigated in *D. melanogaster* for many years and several studies have confirmed the genetic plasticity of this behavior (Takamura and Fuyama, 1980; Allemand and Boulétreau-Merle, 1989; Yang et al., 2008; Miller et al., 2011). *D. melanogaster* is a generalist insect, keeping a broad possibility to feed, lay eggs and develop. Even after being reared for decades in laboratories, the species still displays oviposition behavioral plasticity that can account for its worldwide distribution. Females did not change their oviposition preference even after being ‘forcibly’ raised on a particular medium for many generations (Abed-Vieillard et al., 2014; Soto et al., 2014). However, in several studies, *D. melanogaster* rapidly responded to selection for oviposition site preference (Markow and O’Grady, 2008 and references therein). Since divergent results were obtained in menthol “forced” line and “free-choice” lines for oviposition and mating, choosing freely the egg-laying sites (as in natural populations) appeared to induce faster changes. Neural pathways underlying the change of oviposition response are under investigation (Joseph and Heberlein, 2012; Zhang et al., 2013). We did not explain why “forced” lines respond differently than the “choice” line to a long term-exposition to menthol. It seems that the possibility of choice decision makes the difference (Brembs, 2011). Further investigations on mechanisms of menthol perception and on the neural pathway involved in the behavioral change induced by menthol in the choice procedure will be of interest.

## Author Contributions

DA-V and JC performed the experiments; DA-V analyzed the data and wrote the paper.

## Conflict of Interest Statement

The authors declare that the research was conducted in the absence of any commercial or financial relationships that could be construed as a potential conflict of interest.
