# Supplementary material for: When Choice Makes Sense: Menthol Influence on Mating, Oviposition and Fecundity in Drosophila melanogaster
Source: Front Integr Neurosci. 2016 Feb 22;10:5. doi: 10.3389/fnint.2016.00005 (PMC4761970; doi:10.3389/fnint.2016.00005)
Supplement: Supplementary file 1 [file Image_1.PDF]

Supplementary Material

When choice makes sense: menthol influence on mating, oviposition and fecundity in *Drosophila melanogaster*

Dehbia Abed-Vieillard* and Jérôme Cortot

*** Correspondence:** dehbia.abed@u-bourgogne.fr

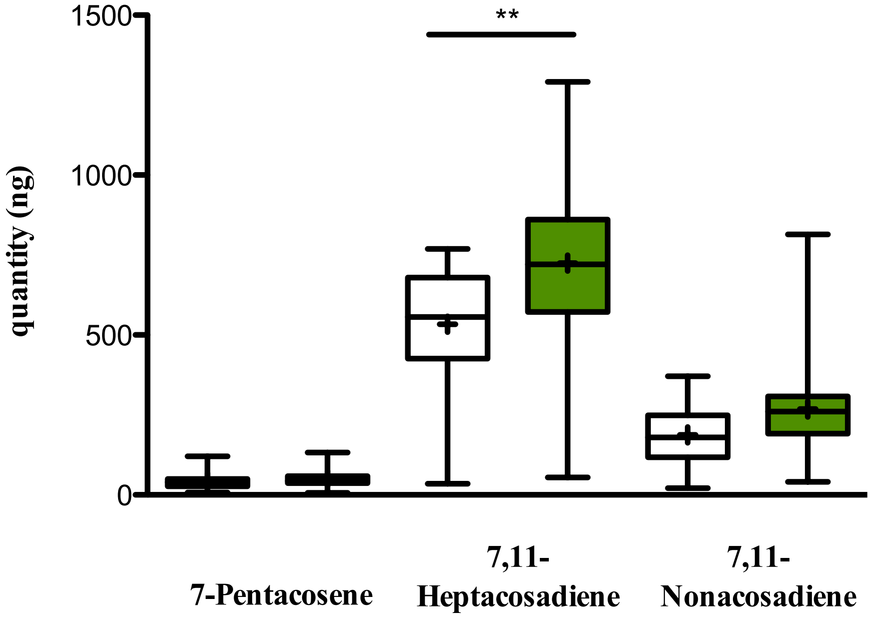

Figure 1: *Drosophila melanogaster* female hydrocarbons.
7,11-heptacosadiene, 7,11-nonacosadiene and 7-pentacosene, the three compounds playing a role in sex recognition, have been quantified by GC in virgin females of the choice-lines.
Data are represented both with their mean (noted by +) and by box-and-whisker plots (the bars represent the first and third quartiles (Q1 and Q3) with the horizontal band inside the bar indicating the median value; the whiskers indicate the minimum and maximum of all of the data.
Quantity in ng of each compound for the P-choice line females (P-line females: open boxes, M-line females : green boxes). ** : $p < 0.01$. (Mann-Whitney test, $p = 0.0083$) N = 16 for P-line females; N = 18 for M-line females.
